# Supplementary material for: All-viral tracing of monosynaptic inputs to single birthdate-defined neurons in the intact brain
Source: Cell Rep Methods. 2022 May 23;2(5):100221. doi: 10.1016/j.crmeth.2022.100221 (PMC9142754; doi:10.1016/j.crmeth.2022.100221)
Supplement: Document S1. Figures S1–S4 and Tables S1 — and S2 [file mmc1.pdf]

Cell Reports Methods, Volume *2*

Supplemental information

All-viral tracing of monosynaptic inputs to single birthdate-defined neurons in the intact brain

R. Irene Jacobsen, Rajeevkumar R. Nair, Horst A. Obenhaus, Flavio Donato, Torstein Slettmoen, May-Britt Moser, and Edvard I. Moser

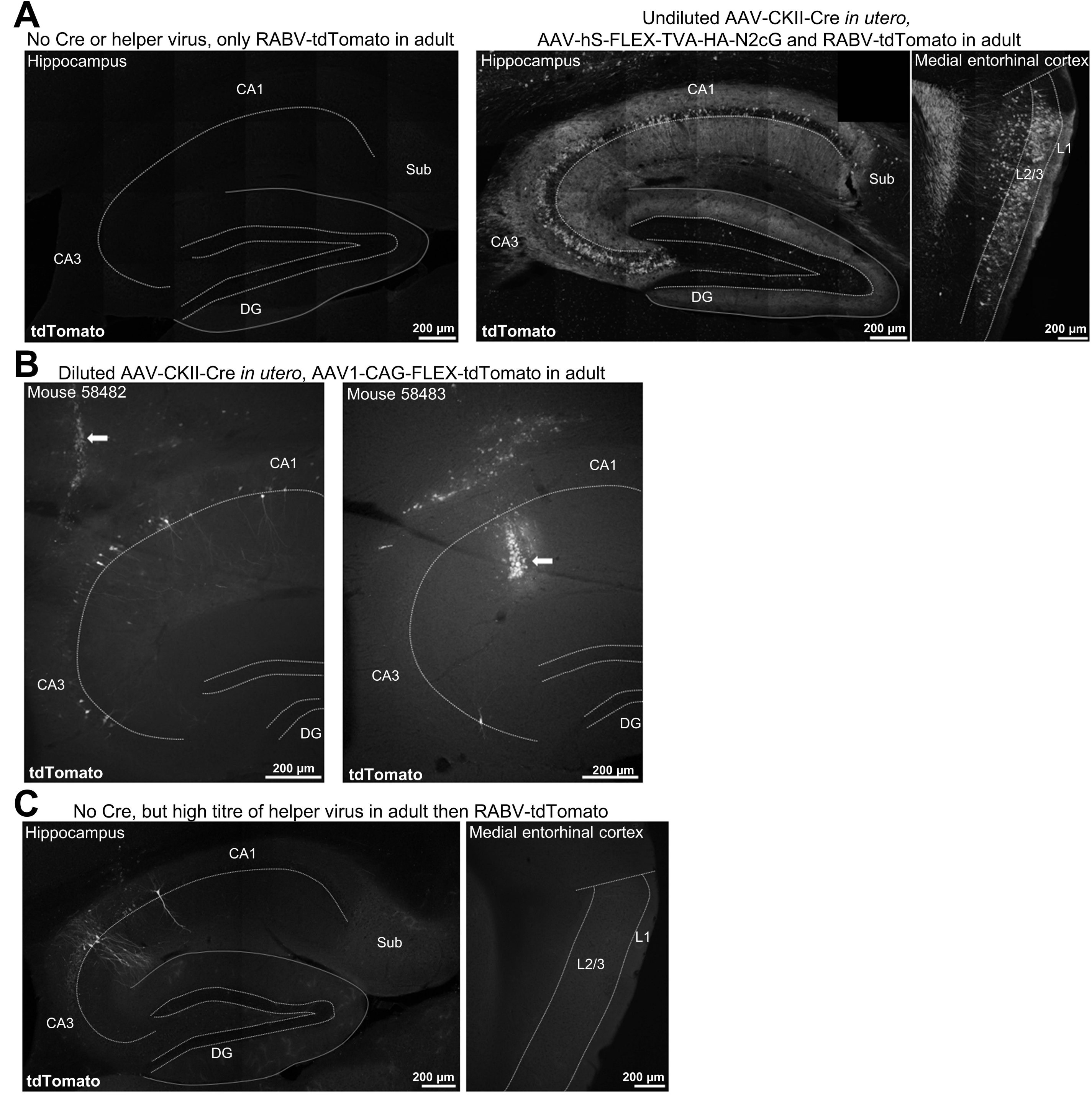

Figure S1: No rabies expression without TVA and G, confirmation of sparse Cre expression, and no spurious rabies expression in MEC, related to figure 1

A) Left: no tdTomato expression in a mouse in which only RABV-tdTomato was injected in the hippocampus. Right: extensive local and distant tdTomato expression in a mouse injected with undiluted AAV-CKII-Cre *in utero* at E12 followed by a large AAV-hS-FLEX-TVA-HA-N2cG injection and separate RABV-tdTomato injection in the adult hippocampus. Images are maximum intensity projections of confocal images. The amount of RABV-tdTomato injected was the same for both mice and they were both perfused 14 days after the rabies injection.

B) Epifluorescence images showing tdTomato expression in two mice that were injected with diluted AAV-CKII-Cre *in utero* at E13 followed by a large (500 nl) AAV1-CAG-FLEX-tdTomato injection in the adult hippocampus with a microliter syringe (#75, Hamilton Company). Arrows indicate parts of the injection track (i.e. not necessarily where the needle was located during the injection itself). Images were acquired as z-stacks and the Extended Focus Module in the Zeiss Zen Blue imaging software used to create a flat image.

C) Epifluorescence images showing a few rabies-expressing cells in the hippocampus but none in the MEC in a mouse without Cre after 138 nl of a high titre (10^{11} infectious particles/ml) helper virus was injected bilaterally into the hippocampus followed by RABV-tdTomato two weeks later. The presence of rabies-expressing cells in the hippocampus is expected given the high helper virus volume and titre used, while the lack of cells in the MEC indicates that Cre-independent expression of TVA in the hippocampus does not impact input labelling in the MEC.

DG: dentate gyrus, Sub: subiculum

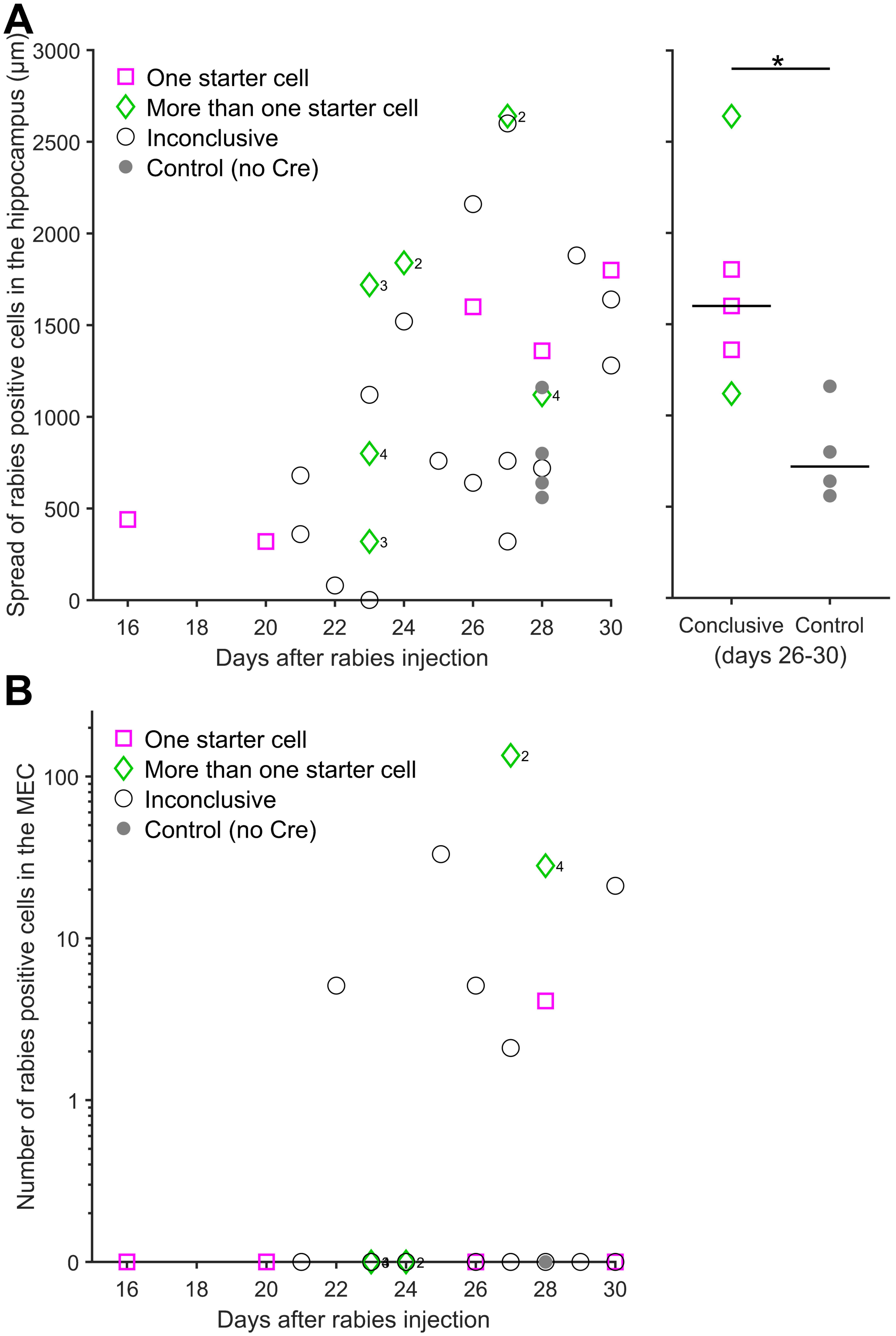

Figure S2: Spread of rabies-positive cells in the hippocampus and number of rabies-positive cells in the MEC, related to figure 2

A) Left: the anatomical spread of rabies-positive cells along the longitudinal axis in the dorsal hippocampus increases as a function of days after the rabies injection ($r = 0.54$, $p = 0.0036$, Spearman's rho, $n = 27$ animals, not including control animals). Right: the spread of rabies-positive cells is larger in conclusive animals compared to control animals, which were all inconclusive ($p = 0.0317$, Wilcoxon rank sum, $n = 5$ conclusive and 4 control animals, black horizontal lines show the median). Spread is calculated based on the number of sections in which rabies-positive cells were observed but does not include the ventral hippocampus (i.e., the spread is likely higher for animals with a long survival time). Animals with zero starter cells are not included as rabies-positive cells are absent.

B) The total number of rabies-positive cells observed in the ipsilateral medial entorhinal cortex (MEC) as a function of days after the rabies injection. Numbers next to diamonds indicate the number of starter cells in the 'More than one starter cell' category. Note: the y-axis follows a log scale and animals with zero starter cells are not included as rabies-positive cells are absent.

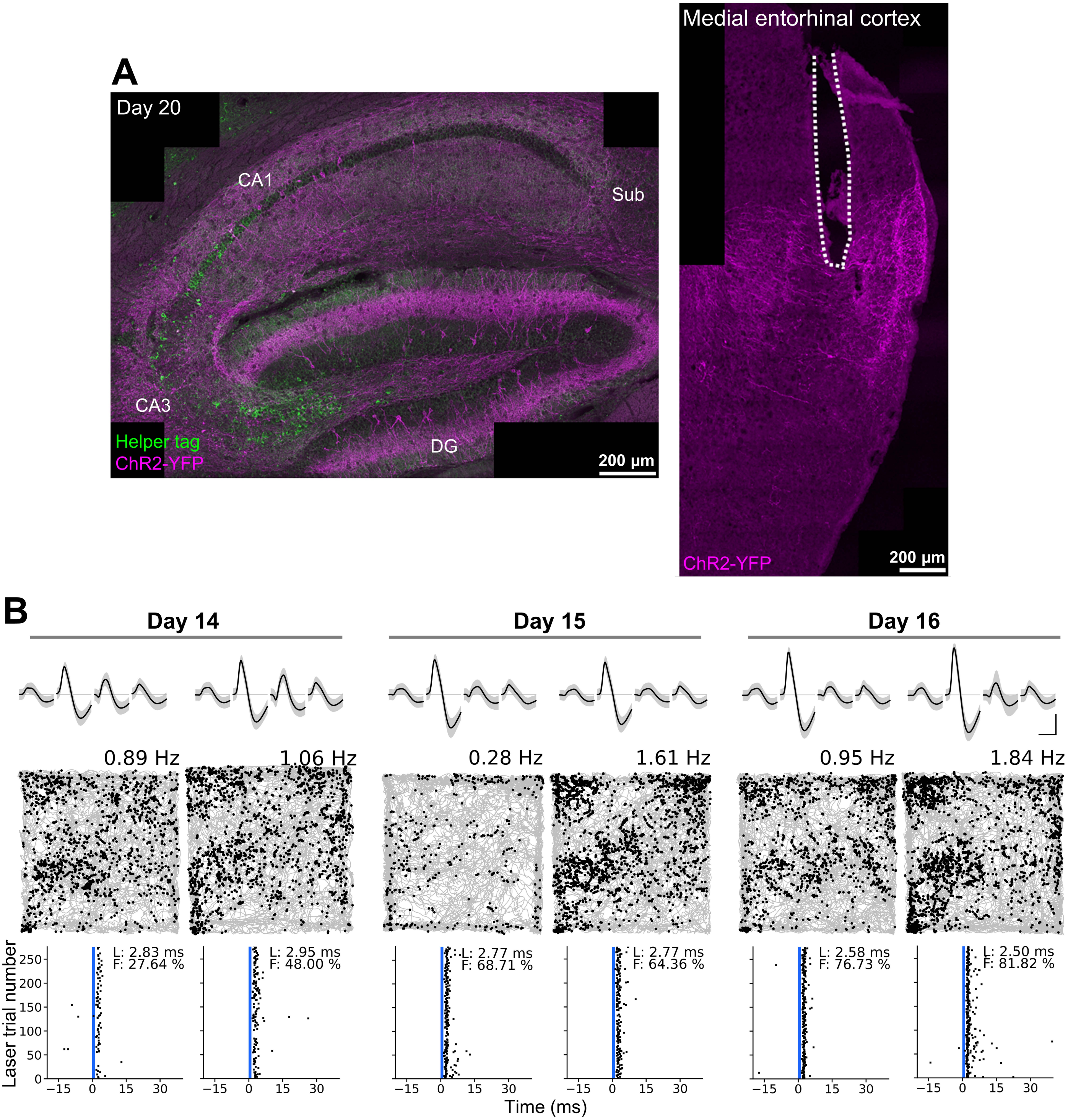

Figure S3: Functional properties of an input cell over time, related to figure 5

A) A large population of starter cells in the hippocampus was targeted by mixing the Cre virus and the helper virus before injection in the adult hippocampus. In a separate, subsequent surgery, a rabies virus carrying ChR2-YFP was injected into the hippocampus, and tetrodes with an optic fibre attached ('optrode') implanted by the ipsilateral MEC (as in **Figure 5**). Left: confocal maximum intensity projection of the hippocampus showing cells expressing helper proteins and/or ChR2-YFP 20 days after the rabies injection. Right: confocal maximum intensity projection showing part of the tetrode track (indicated by white dotted line) and expression of ChR2-YFP in the MEC.

B) Waveforms (top) and path plots (middle) of a cell that was recorded in six different sessions across three days (14, 15, and 16 days after the rabies injection). Subsequent laser trials (1 ms pulses at 10 Hz and 7.5 mW, indicated by vertical blue line) in a separate holding box (as in **Figure 5**) showed fast, robust and reliable responses to laser stimulation (bottom), indicating that this cell expresses ChR2 and provides monosynaptic input to the hippocampus. Grey lines indicate the path of the mouse in the open field while black dots indicate locations (path plots) or time relative to laser stimulation (laser stimulation plots) at which units from the cell was recorded. Scale bars for waveform plots: 0.5 ms (horizontal) and 50 µV (vertical). Values above path plots show the average firing rate in the open field. L: latency, F: fidelity.

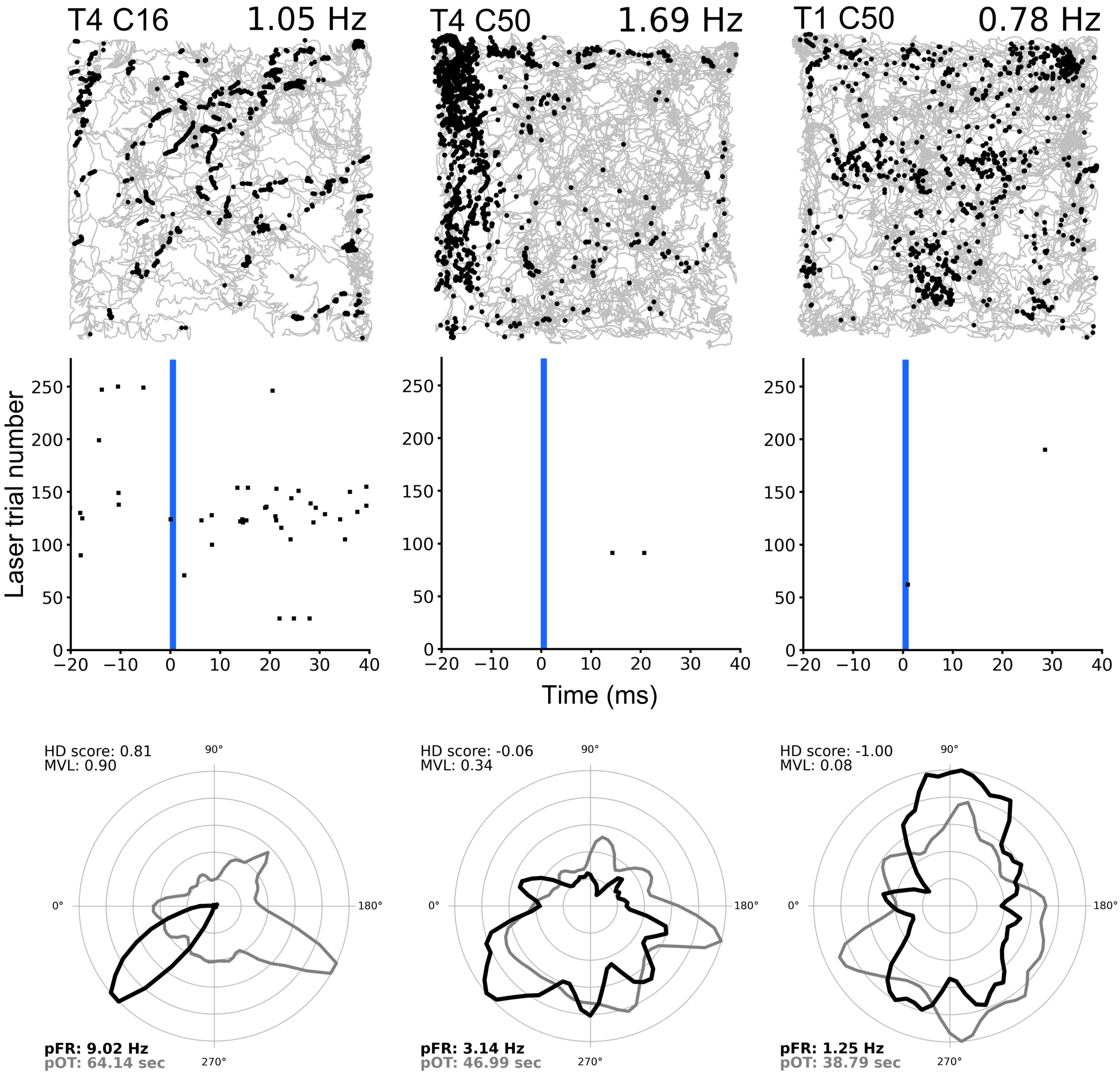

Figure S4: Non-responsive cells, related to figure 5

Path plots (top) of three different example cells recorded in the same area as those depicted in **Figure 5**, but that do not respond to laser stimulation (middle, blue vertical line). Head direction (HD) plots from the open field recordings are also shown (bottom, firing rate (black) and occupancy time (grey) are normalised to their own maximum in each session). The cells shown include a head direction cell (left, 17 days after rabies injection), a border cell (middle, 18 days after rabies injection) and a grid cell (right, 19 days after rabies injection). Grey lines in the open field indicate the path of the mouse while black dots indicate locations (path plots) or time relative to laser stimulation (laser stimulation plots) at which units from an individual cell was recorded. Right-centered values above path plots show the average firing rate in the open field. 1 ms laser pulses were delivered at 10 Hz and with 7.5 mW power. T: tetrode number, C: cell ID, MVL: mean vector length, pFR: peak firing rate, pOT: peak occupancy time.

Animal ID	Sex	Cre dilution	Helper virus (nl)	Days with rabies	Helper tag	Starter cells	Starter cell identity	Rabies cells in hipp	Rabies cells in EC	Figure
**59457**	**M**	**1000**	**41.4**	**16**	**2A**	**1**	**CA3**	**8**	**0**	**2, 3, S2**
**60047**	**F**	**None**	**82.8**	**20**	**2A**	**1**	**GC**	**2**	**0**	**2, 4, S2**
61092	F	500	55.2	21	HA	Inconclusive		2	0	2, S2
61144	M	500	55.2	21	HA	Inconclusive		4	0	2, S2
61159	M	500	55.2	22	HA	0		0	0	2, S2
61164	M	500	55.2	22	HA	Inconclusive		3	5	2, S2
59930	F	500	147.2	23	2A	3	GC, CA, sl	12	0	2, S2
59931	F	500	101.2	23	2A	4	2xGC, CA1, Sub	38	0	2, S2
59944	F	500	46	23	2A	3	2xCA1, CA1sr	11	0	2, S2
59946	F	500	92	23	2A	Inconclusive		30	0	2, S2
59975	M	500	115	23	2A	Inconclusive		1	0	2, S2
60650	F	500	69	24	HA	2	CA, WM	101	0	2, S2
60651	F	500	55.2	24	HA	Inconclusive		33	0	2, S2
59943	M	500	46	25	2A	0		0	0	2, S2
59977	M	500	55.2	25	2A	Inconclusive		32	33	2, S2
59979	F	500	55.2	26	2A	Inconclusive		7	0	2, S2
**60652**	**F**	**500**	**55.2**	**26**	**HA**	**1**	**GC**	**36**	**0**	**2, 4, S2**
60821	M	500	55.2	26	HA	Inconclusive		116	5	2, S2
60222	F	500	55.2	27	2A	Inconclusive		72	0	2, S2
60221	F	500	55.2	27	2A	Inconclusive		50	2	2, S2
60822	M	500	55.2	27	HA	Inconclusive		7	0	2, S2
60823	F	500	55.2	27	HA	2	CA3sr, CA3	668	135	2, S2
**60259**	**F**	**500**	**55.2**	**28**	**HA**	**1**	**DG**	**35**	**4**	**2, 4, S2**
60824	F	500	55.2	28	HA	4	GC, CA3, WM, CA1so	74	28	2, S2
60825	F	500	55.2	28	HA	Inconclusive		54	0	2, S2
60218	M	500	55.2	29	2A	0		0	0	2, S2
60919	F	500	55.2	29	HA	Inconclusive		8	0	2, S2
60920	F	500	55.2	29	HA	0		0	0	2, S2
**60219**	**M**	**500**	**55.2**	**30**	**2A**	**1**	**CA so**	**27**	**0**	**2, 3, S2**
60389	M	500	55.2	30	2A	Inconclusive		74	21	2, S2
60390	M	500	69	30	HA	Inconclusive		97	0	2, S2

Table S1: Experimental details and analysis of animals used for single cell experiments, related to Figures 2, 3, 4, S2, and STAR Methods

All animals were injected with Cre at E13 and animals with a single starter cell are highlighted in bold. The number of rabies positive cells in the dorsal hippocampus includes starter cells. The table is sorted by the ‘Days with rabies’ column. All CA/CA3/CA2/CA1 cells are in the pyramidal cell layer unless indicated to be in so or sr.

hipp: dorsal hippocampus, EC: entorhinal cortex, CA: cell in the CA3/CA2/CA1 border area, GC: granule cell, Sub: cell in the subiculum, WM: cell in the white matter, sl: cell in stratum lucidum, so: cell in stratum oriens, sr: cell in stratum radiatum

Animal ID	Sex	Embryonic day for Cre injection	Cre dilution	Helper virus (nl)	Days with rabies	Helper tag	Experiment	Figure
72359	M	N/A	N/A	N/A	14	N/A	WT with rabies	S1
58695	M	E12	None	404.8	14	N/A	Cre, N2cG and rabies	S1
58482	M	E13	500	N/A	N/A	N/A	Check of Cre expression	S1
58483	M	E13	500	N/A	N/A	N/A	Check of Cre expression	S1
79986	F	N/A	N/A	138.0	14	N/A	WT with high titre N2cG	S1
99864	F	N/A	N/A	55.2	28	HA	WT with N2cG and rabies	2, S2
99584	F	N/A	N/A	55.2	28	HA	WT with N2cG and rabies	2, S2
99877	M	N/A	N/A	55.2	28	HA	WT with N2cG and rabies	2, S2
99878	M	N/A	N/A	55.2	28	HA	WT with N2cG and rabies	2, S2
70375	M	Adult Cre injection	N/A	151.8	20	2A	Optogenetics	S3
58313	F	E12	None	404.8	30	2A	Optogenetics	5, S4
58253	F	E14	None	404.8	24	2A	Ca^{2+} imaging	6
60388	M	E13	500	55.2	34	2A	Ca^{2+} imaging	7

Table S2: Experimental details of animals used for control and functional experiments, related to Figure 2, 5, 6, 7 S1, S2, S3, S4, and STAR Methods

WT: wildtype (i.e., a mouse not injected with Cre *in utero*).
